# The relationship between attentional control and injury-related biomechanics in young female volleyball players

**DOI:** 10.3389/fphys.2025.1622026

**Published:** 2025-07-04

**Authors:** Ivana Hanzlíková, Karolína Válová, Michal Lehnert, Martin Dvořáček, Elisa Doleželová, Adam Grinberg

**Affiliations:** ^1^ Department of Physiotherapy, Faculty of Physical Culture, Palacký University Olomouc, Olomouc, Czechia; ^2^ Department of Sport, Faculty of Physical Culture, Palacký University Olomouc, Olomouc, Czechia; ^3^ Department of Community Medicine and Rehabilitation, Umeå University, Umeå, Sweden

**Keywords:** Flanker test, response inhibition, reaction time, LESS, dynamic balance, stiffness, reactive strength, biomechanical risk factors

## Abstract

**Background:**

Adolescent athletes, particularly in team sports, exhibit high risk of non-contact injuries due to the open environment and risk-associated movements. Both biomechanical risk factors and suboptimal neurocognitive function have been linked to such injuries. The association particularly between attentional control and injury-related biomechanics remains unexplored in young athletes.

**Methods:**

Fifty female volleyball players aged 7–15 years participated. Attentional control was assessed using the Eriksen Flanker test (congruent, incongruent reaction times (RT) and interference effect). Biomechanical measures included the Landing Error Scoring System (LESS), single-leg dynamic balance (center of pressure [CoP] movement), leg stiffness during submaximal hopping, and reactive strength index (RSI) during drop jumps. Spearman’s rank correlation and partial Spearman’s rank correlation (controlling for age) were used.

**Results:**

When controlling for age, a moderate positive correlation was observed between the Flanker interference effect and CoP movement in the antero-posterior direction of the non-dominant leg (r_s_ = 0.40, *r* ^2^ = 0.16). When age was not accounted for, additional moderate negative correlations were observed between congruent and incongruent reaction times and leg stiffness, as well as with RSI.

**Conclusion:**

While response inhibition was positively associated with dynamic balance, other biomechanical measures, seemed to follow a more age-dependent developmental trajectory. Among injury-related biomechanical risks, only dynamic balance can thus be considered more related to neurocognitive function. Sport practitioners are advised to consider coupling dynamic stability exercises with neurocognitive evaluations for more holistic prevention of injuries in young athletes.

## Introduction

Team sports such as volleyball often demand not only physical but also mental performance, including rapid decision-making, reactions to the environment and good neuromuscular coordination (Myer et al., 2011). Athletes are constantly exposed to changing conditions during play, requiring quick and appropriate responses as well as inhibition to inappropriate ones. An interplay between these neurocognitive functions and motor skills is critical for both optimal performance and for reducing injury risk (Hughes and Dai, 2021; Bertozzi et al., 2023). During cognitively demanding tasks, athletes may be at a higher risk of injury due to suboptimal motor planning, increased attentional demands, poor neuromuscular control–the ability to coordinate muscle activation for movement and joint stability–and impaired coordination, all of which can lead to injury-prone movement patterns (Giesche et al., 2018; Piskin et al., 2022; Bertozzi et al., 2023). Moreover, open-skill sports that include elements of reaction time and decision-making typically involve a higher presence of risky biomechanical patterns (Almonroeder et al., 2018; Avedesian et al., 2022). For example, increased dynamic valgus of the knee joint is suggested to be associated with the risk of lower limb injuries due to long-term non-optimal loading of musculoskeletal structures with gradual overloading during more demanding movements (Shin et al., 2009; Tamura et al., 2017). Injuries often occur during fast movements which require significant neuromuscular control, such as landing from jumps and changes in direction or speed (Piskin et al., 2022; Bertozzi et al., 2023).

The risk for non-contact injuries is particularly large for adolescent athletes (Bram et al., 2021), with both musculoskeletal growth and motor control developing gradually during adolescence, potentially leading to non-optimal neuromuscular control and abnormal biomechanics (Hewett et al., 2004; Ford et al., 2010; Myer et al., 2011). During this period, biological maturation occurs alongside chronological age but may follow a non-linear trajectory (Cobley et al., 2025). This maturation process is associated with differences in sport performance (Cobley et al., 2025) and, more notably, with increased injury risk (Materne et al., 2021). The risk is further enhanced among adolescent girls who tend to show worse neuromuscular control, less muscle strength, and differences in musculoskeletal development, such as wider pelvises and more frequent knee valgus positions compared to boys (Arendt and Dick, 1995; Brent et al., 2008; Beutler et al., 2009).

One of the commonly used clinical tools to evaluate biomechanical risk for injury is the Landing Error Scoring System (LESS), which assesses 17 kinematic errors related to landing positions associated with Anterior cruciate ligament (ACL) loading (Padua et al., 2009). It was deemed suitable for applications in team sports due to its minimal requirements of space, time and equipment. It has also demonstrated high reliability, including intrarater, interrater and intersession reliability, with individual item validity related to ACL injury risk factors ranging from moderate to excellent (Hanzlíková and Hébert-Losier, 2020). Previous work reported higher (worse) LESS scores among females compared to males (Beutler et al., 2009; Padua et al., 2009), with females demonstrating distinctly different kinematics, such as reduced hip and knee flexion at initial contact, increased knee valgus with a wider stance and less knee-flexion displacement (Beutler et al., 2009).

Worse LESS scores have also been demonstrated among younger individuals (14–18 years) compared to older participants (18–23 years) (Smith et al., 2012). Collectively, the literature supports the greater biomechanical risk of injury which characterizes both females (Hewett et al., 2004; Beutler et al., 2009; Fox et al., 2014) and adolescents (Smith et al., 2012; Bram et al., 2021), a risk that can be reflected in higher LESS scores.

A second measure related to injury risk is dynamic balance, the ability to maintain postural stability during active movements (O'Sullivan et al., 2019). It has been established extensively (Hrysomallis, 2007) that deficient dynamic balance is associated with greater occurrence of lower-limb injuries. Moreover, balance training programs were shown to be effective at reducing injury risk (Emery et al., 2005; Yan et al., 2025), possibly through neuromuscular mechanisms, affecting muscle co-contraction during dynamic tasks (Lloyd, 2001; Gatts and Woollacott, 2006). It has further been suggested that balance training has a positive effect on knee sagittal-plane kinematics, improving force dissipation during single-leg drop landings (Myer et al., 2006).

Finally, two additional measures associated with injury risk are reactive strength and leg stiffness, both were found valid and reliable for use among youth athletes (Dalleau et al., 2004; Flanagan and Comyns, 2008; Lloyd et al., 2009). They are considered indicators of neuromuscular mechanisms particularly important for functional stability involving the stretch-shortening cycle (SSC) (Toumi et al., 2006; Raschner et al., 2012). In previous studies, the RSI has been described as the capability of the low extremity muscles to change quickly from an eccentric to concentric contraction involved in SSC and as a tool to monitor stress on the muscle-tendon complex during exercises with SSC (Young, 1995; Flanagan and Comyns, 2008; Lloyd et al., 2009). Low values of RSI indicate a lower ability to tolerate impact forces and are considered a potential risk factor in ACL injury (Young, 1995; Jarvis et al., 2022). Leg stiffness points to an ability to generate strength and resist to deformation resulting from movement including a direct transition from eccentric to concentric muscle contraction (Lloyd et al., 2009).

While evidence exists demonstrating that worse cognitive performance is associated with injury biomechanical risk profile (Avedesian et al., 2022; Bertozzi et al., 2023), this association has not yet been assessed in a younger population. The nature of such a relationship can theoretically be related to injury risk in younger age groups, given that abilities such as reaction time and attentional control have been shown to be strongly dependent on maturation level (Grinberg et al., 2024).

The aim of this study was to assess the association between attentional control, measured by the Eriksen’s Flanker test, and injury-related biomechanics, including LESS performance, dynamic balance, reactive strength and lower limb stiffness, in young female volleyball players. We hypothesized strong correlations even when controlling for age, suggesting that in this injury-prone population, a strong relationship between neurocognitive and biomechanical performance exists and warrants the attention of sport practitioners when designing training and injury-prevention interventions. Supplementary analyses controlling for maturation status were also conducted to confirm the findings given the non-linearity of biological maturation in this age group.

## Materials and methods

### Participants

To detect a moderate correlation of 0.40, as specified by Akoglu (2018) (Akoglu, 2018), we calculated the required sample size using G*Power version 3.1.9.7. This estimation, based on standard two-tailed hypothesis testing with an 80% power (β = 0.20) and a 5% significance level (α = 0.05), indicated a required sample size of 44 participants.

From an initial pool of 80 potential participants, 56 agreed to take part, but only 50 were included in the analysis due to missing data or task performance issues. All participants were female volleyball players aged 7 to 15 from the same club, with a training frequency of three times per week. We ensured that none had injuries or pain affecting their activity and excluded those with significant injuries or surgeries in the past 3 months, as well as those with diagnosed neurodevelopmental disorders (e.g., ADHD, autism spectrum disorders).

The study protocol was approved by the health research ethics committee at Palacký University Olomouc (15/2023) and adhered to the Declaration of Helsinki. Written informed consent was obtained from the guardians of all participants.

#### Procedure

Assessments were conducted over two consecutive days. On the first day, participants’ height, weight, dynamic balance and the Flanker test were measured. On the second day, the rest of the biomechanical measurements were taken–LESS, submaximal hopping to calculate leg stiffness and drop jumps to assess their reactive strength index (RSI).

A standardized 10-min warm-up was conducted before each session, consisting of dynamic stretching, bilateral and unilateral vertical submaximal jumps, horizontal jumps, and three progressive sprints at 60%, 90%, and 100% of maximum effort. At the start of each session, participants were divided into groups of three and rotated through stations in a randomized order while wearing sports attire and their own shoes. For all measurements, the dominant leg was defined as the leg typically used to kick a ball. To minimize order effects, the order of testing each leg for balance and leg stiffness assessments was randomized. All participants were unfamiliar with the tasks and received standardized instructions along with equal practice trials to minimize learning effects.

#### Eriksen Flanker test

The Flanker test was utilized in this study to assess attentional control and response inhibition, selected for its established reliability and suitability for young female participants (Rueda et al., 2004). To ensure an optimal testing environment: The chair height was adjusted to align the table with the participants’ elbow level, and a footrest was used to stabilize their feet if necessary. The computer screen was set at arm’s length and adjusted so the top row of letters matched the participants’ eye level.

Participants were directed to sit comfortably with their left index finger on the “A” key and their right index finger on the “L” key, awaiting the start of the test. They received standardized instructions explaining that they would respond to one of four letters. Specifically, the letters X and C required pressing the “A” key, while V and B required the “L” key. Participants were instructed to focus on the central letter while ignoring surrounding letters, which could be either congruent (i.e., requiring a similar action as the central letter) or incongruent (i.e., requiring a different response than the central letter). A practice trial was conducted to ensure understanding, continuing until ten correct responses were made. Headphones were then provided to reduce external noise, and the examiner remained out of the participants’ line of sight to minimize any observer effects. Each participant performed a total of 50 trials.

For statistical analysis, congruent and incongruent reaction times (ms), as well as the Flanker interference effect, were calculated. The Flanker interference effect, a measure for response inhibition, was defined as the difference between average incongruent and congruent reaction times, with positive value indicating slower reaction times for incongruent trials compared to congruent ones.

#### Landing error scoring system (LESS)

For evaluating the risk of non-contact injuries related to neuromuscular control and biomechanics, the LESS procedure was used (Padua et al., 2009). The testing equipment included a 30 cm high box and a marked landing zone on the ground, with the distance from the box to the landing zone set at 50% of the participant’s height. Each participant completed the test wearing their standard volleyball training shoes. They were instructed to jump from the box onto the landing zone and then perform a maximal vertical jump. Each participant received one practice trial before performing three recorded trials, with a minimum of 30 s rest between trials. If a participant showed signs of fatigue, additional rest was provided to ensure readiness for subsequent trials. Two digital cameras (SONY HXR-MC2000 and SONY HXR-NX5E) with a 50 Hz capture rate were used for recording. Cameras were positioned 1.2 m above the ground and 3 m from the landing zone, with one camera capturing the frontal plane and the other capturing the sagittal plane. Footage from all the trials was analyzed using Kinovea® (version 0.9.5) in conjunction with the LESS scoring sheet (Padua et al., 2009). Each video was randomly assigned to three raters for evaluation. To assess intra-rater reliability, an intraclass correlation coefficient (ICC_(3,1)_) of 0.86 (95% confidence intervals = 0.68–0.95) was calculated for the first 10 participants, indicating strong inter-rater reliability. The average LESS score from the three trials for each participant was used for statistical analysis.

#### Single-leg dynamic balance

To assess dynamic balance, participants performed a single-leg squat test on a force platform (9260AA6, Kistler Group, Winterthur, Switzerland; sampling rate of 200 Hz), positioned 2 m from a wall and following the protocol outlined by (Culvenor et al., 2016).

Participants stood barefoot on one leg in the middle of the platform, with their non-supporting leg extended forward and arms crossed over their chest. They focused on a vertical strip of tape placed on the wall in front of them. A horizontal bar was placed behind the participant, aligned with their heels, to indicate the desired squat depth (i.e., the bar touched the participant’s buttocks upon reaching the target depth). This depth corresponded to 60° of knee flexion, which was confirmed using a goniometer prior to testing. Participants were instructed to complete five single-leg squats to the specified depth, timed with a metronome set at 0.5 Hz, allowing 2 s for both the descent and ascent phases of each squat. No explicit instructions were provided for trunk posture. A practice trial was conducted to familiarize participants with the task. Trials were considered invalid if the non-supporting leg touched the ground, if the arms were moved or if the participant sat on the bar. Invalid trials were repeated. Outcome variables included mean speed and range of motion (ROM) of the center of pressure (CoP) in both the antero-posterior and medio-lateral directions, for each leg. Lower values indicated better dynamic balance (Prieto et al., 1996).

#### Leg stiffness

Sub-maximal hopping was employed to calculate leg stiffness, with contact and flight times collected using a mobile contact mat connected to an electronic hub (SmartJump, Fusion Sport, Brisbane, Australia). Leg stiffness was determined using the following formula (Dalleau et al., 2004):

$$Stiffness=\frac{M\times \pi \left(Tf+Tc\right)}{{Tc}^{2}\left(\frac{Tf+Tc}{\pi }-\frac{Tc}{4}\right)}$$

where M is total body mass, Tc the ground contact time and Tf the flight time.

This method was shown to be valid and reliable for youth athletes on a contact mat (Dalleau et al., 2004). Participants began with bilateral hopping at frequencies of 2.2 Hz, followed by unilateral hopping assessed at 2.5 Hz on both the dominant and non-dominant legs (Lloyd et al., 2009; Beerse and Wu, 2016). Each participant completed one trial of 20 continuous hops, guided by sound of a digital metronome (Eiling et al., 2007; Lloyd et al., 2009). To minimize fatigue, a 3-min rest period was provided between hop types. Participants were instructed to keep their hands on their hips, land in the same spot, land with legs extended, and focus their gaze forward to reduce arm and trunk influence (Lloyd et al., 2009). The average stiffness from all valid contacts was used for analysis. Trials in which the rhythm was lost, participants jumped off the mat, or exceeded 300 m contact time were excluded.

#### Reactive strength index (RSI)

The RSI is a common measure of an athlete’s explosiveness, reflecting their ability to generate maximal force in minimal time during movements that utilize the stretch-shortening cycle (SSC) (Zatsiorsky et al., 2020). It was obtained using a 30 cm drop-jump test with the *Opto-jump Next* system (Microgate, Bolzano, Italy). Participants stepped off the platform with hands on hips, maintaining a forward gaze, and aimed to minimize ground contact time before jumping as high as possible (Flanagan and Comyns, 2008). They were instructed to keep their legs extended during the jump, avoiding leg-tucking. Trials in which participants stepped down improperly were excluded and repeated. Three successive trials were conducted, with the average of the best two used for analysis (Sole et al., 2007). Resting period between attempts was 30 s. The RSI was calculated as the ratio of jump height to contact time, a method shown to be reliable in youth athletes (Flanagan and Comyns, 2008; Lloyd et al., 2009).

#### Statistical analysis

Data normality was evaluated using the Shapiro-Wilk test. Descriptive statistics were reported as mean ± standard deviation for normally distributed data or median (interquartile range) for non-normally distributed data.

To determine correlations, Spearman’s rank correlation tests were used. In order to account for potential confounding effect of age without introducing model complexity or stratification in a relatively small sample, partial Spearman’s rank correlations were performed, with chronological age included as a covariate. To verify the assumption of monotonicity required for correlations, scatterplots of the relevant variable pairs were generated and visually assessed. Additionally, as further validation (Supplementary Material), we conducted partial Spearman’s rank correlations controlling for the percentage of predicted adult height–estimated using the Khamis-Roche method (Khamis and Roche, 1994)–as a proxy for biological maturation. Correlation coefficients were interpreted based on the following thresholds: 0–0.39 for weak; 0.40–0.69 for moderate; 0.70–0.99 for strong; and 1 for perfect correlation (Akoglu, 2018). In addition, we calculated the coefficient of determination (*r* ^*2*^) for significant results to estimate the proportion of variance in the outcome variable explained by each predictor. A total of 78 correlations were tested, as each biomechanical variable was correlated with each Flanker performance outcome (congruent RT, incongruent RT, and Flanker interference effect), resulting in 39 original and 39 partial Spearman correlations. To control for multiple comparisons, false discovery rate (FDR) adjustments were made using the Benjamini–Hochberg procedure (Benjamini and Hochberg, 1995). Data were analyzed using RStudio® version 2023.09.02 and Microsoft Excel® MS Office 365. Statistical significance was set at α < 0.05.

## Results

### Participants’ descriptive information

A sample of 50 female volleyball players was analyzed. Age, % of predicted adult height, years of active volleyball training, height, mass, and body mass index (mean ± standard deviation) were 12.32 ± 2.04 years, 91.48% ± 7.76%, 2.6 ± 1.8 years, 158.37 ± 12.34 cm, 50.21 ± 13.22 kg, and 19.71 ± 3.51 kg/m^2^, respectively. Medians with interquartile range (IQR), along with the minimum and maximum values for each measured variable, are presented in Table 1.

**TABLE 1:** Descriptive statistics of measured variables.

Variables	Median (IQR)	Min to max
Flanker interference effect (ms)	42.5 (78)	−129 to 308
Congruent RT (ms)	732 (195.5)	492 to 1414
Incongruent RT (ms)	780 (219)	553 to 1360
LESS (errors)	5.7 (1.8)	3.33 to 11
Balance dominant leg		
ROM CoP ML (cm)	4.1 (0.9)	2.7 to 10.9
ROM CoP AP (cm)	6.5 (3.0)	4.7 to 14.6
Speed CoP ML (cm/s)	4.7 (1.4)	3.3 to 7.1
Speed CoP AP (cm/s)	6.3 (1.5)	4.1 to 9.5
Balance non-dominant leg		
ROM CoP ML (cm)	3.8 (1.0)	2.7 to 7.4
ROM CoP AP (cm)	6.9 (1.4)	4.4 to 14.3
Speed CoP ML (cm/s)	4.5 (1.2)	2.9 to 7.7
Speed CoP AP (cm/s)	6.2 (1.7)	4.2 to 9.0
Bilateral Leg Stiffness 2.2 Hz (kN/m)	19.6 (6.3)	10.2 to 34.9
Dominant Leg Stiffness 2.5 Hz (kN/m)	16.5 (5.7)	8.7 to 23.8
Nondominant LS 2.5 Hz (kN/m)	17.2 (5.7)	8.7 to 23.6
Reactive Strength Index, RSI (m/s)	0.9 (0.4)	0.4 to 1.8

AP, antero-posterior direction; CoP, center of pressure; IQR, interquartile range; LESS, landing error scoring system; Min to max, minimal to maximal values; ML, medio-lateral direction; ROM, range of motion; RT, reaction time.

### Correlations, not controlling for age

**FIGURE 1:**
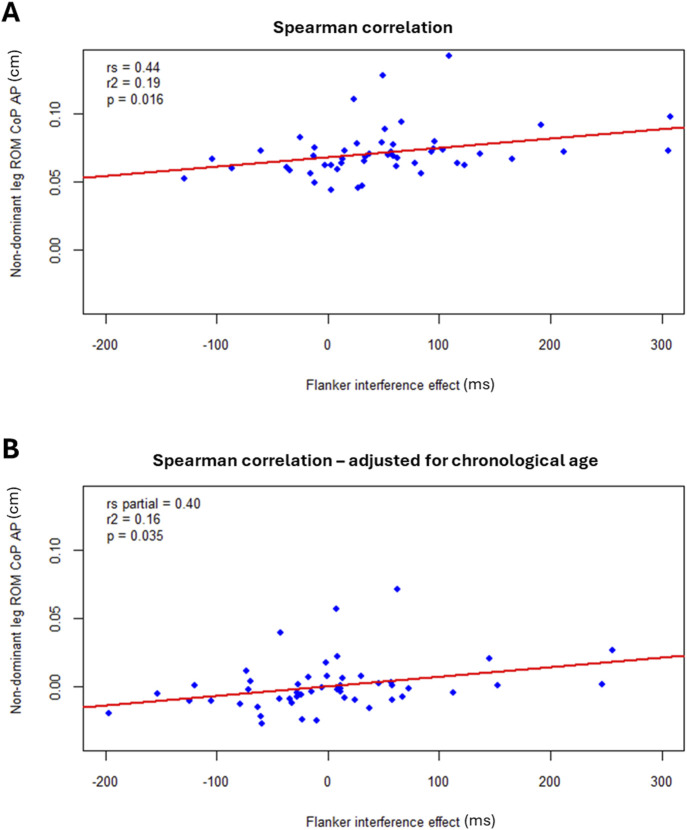
Correlations between Flanker interference effect and range of motion (ROM) of the center of pressure (CoP) of the non-dominant leg, in the antero-posterior (AP) direction. **(A)** Unadjusted correlation. **(B)** Partial correlations, controlling for chronological age (residual plot).

Moderate positive correlation was found between the Flanker interference effect and the range of CoP movement in the antero-posterior direction during dynamic balance on the non-dominant leg (r_s_ = 0.44, *r* ^2^ = 0.194, P = 0.016, Table 2; Figure 1A). Moderate negative correlations were observed between both congruent and incongruent reaction times and all leg stiffness measures (r_s_ = −0.39 to −0,50, *r* ^2^ = −0.152 to −0.250 P < 0.047, Table 2). Additionally, moderate negative correlations were found between both congruent and incongruent RT and RSI (r_s_ = −0.54 and −0.56, *r* ^2^ = −0,29 and −0.31, P = 0.006 and P = 0004, respectively; Table 2).

**TABLE 2:** Spearman´s rank correlation between Flanker test performance and risk factors–Not controlling for age.

Correlated variables	Congruent RT		Incongruent RT T		Flanker interference effect	
	r_s_	P-value (FDR)	r_s_	P-value (FDR)	r_s_	P-value (FDR)
LESS (errors)	0.14	0.748	0.14	0.748	0.13	0.748
Balance dominant leg						
ROM CoP ML (cm)	−0.07	0.813	−0.03	0.951	0.14	0.748
ROM CoP AP (cm)	0.23	0.386	0.31	0.150	0.34	0.085
Speed CoP ML (cm/s)	−0.02	0.951	−0.05	0.901	0.03	0.950
Speed CoP AP (cm/s)	0.16	0.713	0.16	0.713	0.11	0.763
Balance non-dominant leg						
ROM CoP ML (cm)	−0.09	0.791	0.02	0.961	0.28	0.251
ROM CoP AP (cm)	−0.01	0.961	0.12	0.748	**0.44**	**0.016**
Speed CoP ML (cm/s)	−0.05	0.912	−0.02	0.951	0.10	0.763
Speed CoP AP (cm/s)	0.20	0.507	0.25	0.307	0.22	0.394
Bilateral Leg Stiffness 2.2 Hz (kN/m)	**−0.50**	**0.006**	**−0.41**	**0.033**	0.01	0.961
Dominant Leg Stiffness 2.5 Hz (kN/m)	**−0.44**	**0.026**	**−0.39**	**0.047**	−0.03	0.950
Nondominant Leg Stiffness 2.5 Hz (kN/m)	**−0.44**	**0.016**	**−0.41**	**0.035**	−0.09	0.791
Reactive Strength Index (RSI)	**−0.54**	**0.006**	**−0.56**	**0.004**	−0.29	0.255

AP, antero-posterior direction; CoP, center of pressure; LESS, landing error scoring system; ML, medio-lateral direction; P-value (FDR), adjusted P values using False Discovery Rate correction; ROM, range of motion; r_s_, Spearman correlation coefficient; RT, reaction time. Significant correlation coefficients are marked in bold (as well as their adjusted p-values).

### Correlations, controlling for age

When accounting for chronological age, the only significant partial correlation was observed between the Flanker interference effect and the range of CoP movement in the antero-posterior direction during dynamic balance on the non-dominant leg (r_s_ partial = 0.40, *r* ^2^ = 0.160, P = 0.035, Table 3; Figure 1B). Similar results were observed when controlling for biological age (Supplementary Table S2).

**TABLE 3:** Partial Spearman´s rank correlation between Flanker test performance and risk factors controlling for age.

Correlated variables	Congruent RT		Incongruent RT		Flanker interference effect	
	r_s partial_	P-value (FDR)	r_s partial_	P-value (FDR)	r_s partial_	P-value (FDR)
LESS (errors)	0.09	0.791	0.09	0.791	0.12	0.748
Balance dominant leg						
ROM CoP ML (cm)	−0.11	0.762	−0.07	0.813	0.11	0.763
ROM CoP AP (cm)	0.14	0.748	0.26	0.328	0.33	0.143
Speed CoP ML (cm/s)	−0.08	0.791	−0.11	0.763	0.02	0.953
Speed CoP AP (cm/s)	−0.03	0.950	−0.00	0.989	0.12	0.748
Balance non-dominant leg						
ROM CoP ML (cm)	−0.11	0.763	0.03	0.950	0.27	0.307
ROM CoP AP (cm)	−0.07	0.813	0.13	0.748	**0.40**	**0.035**
Speed CoP ML (cm/s)	−0.11	0.763	−0.06	0.898	0.14	0.748
Speed CoP AP (cm/s)	0.01	0.961	0.13	0.748	0.25	0.328
Bilateral Leg Stiffness 2.2 Hz (kN/m)	0.03	0.951	0.15	0.748	0.15	0.748
Dominant Leg Stiffness 2.5 Hz (kN/m)	0.17	0.713	0.20	0.515	0.08	0.809
Nondominant Leg Stiffness 2.5 Hz (kN/m)	0.08	0.791	0.10	0.763	−0.01	0.989
Reactive Strength Index (RSI)	−0.15	0.748	−0.22	0.454	−0.24	0.386

AP, antero-posterior direction; CoP, center of pressure; LESS, landing error scoring system; ML, medio-lateral direction; P-value (FDR), adjusted P values using False Discovery Rate correction; ROM, range of motion; r_s_ partial. Partial Spearman correlation coefficient (accounting for age) RT, reaction time. Significant correlation coefficients are marked in bold (as well as their adjusted p-values).

A supplementary analysis (Supplementary Table S1) confirmed strong positive correlations between chronological age and bilateral, dominant leg and non-dominant leg stiffness (r_s_ = 0.71 to 0.74, P < 0.001) and moderate positive correlation between chronological age and RSI (r_s_ = 0.66, P < 0.001). In addition, moderate negative correlations were observed between chronological age and both congruent (r_s_ = −0.69, P < 0.001) and incongruent RT (r_s_ = −0.66, P < 0.001).

## Discussion

The overall objective of this study was to explore the relationship between attentional control and injury-related biomechanics in young female volleyball players. Our findings indicate significant correlations between attentional control and injury-related biomechanics when age was not accounted for. However, when controlling for age, the only remaining significant association was between Flanker interference effect and antero-posterior CoP movement of the non-dominant leg.

Both attentional control (Salthouse, 2010; Grinberg et al., 2024) and biomechanics (Westbrook et al., 2020) are known to improve with age due to natural development and training. As children grow older, their cognitive and physical abilities mature, which might explain the correlations observed when age was not controlled for. Particularly for leg stiffness and reactive strength index, a supplementary analysis (Supplementary Table S1) confirmed their significant correlations with age. These can be explained by multiple changes in the developing organism. Previous literature identified key neuromuscular and structural changes in growth and maturity which positively influence the SSC capability. It was suggested that because of the development of intrinsic neuromuscular properties during childhood, neural regulation of SSC becomes more efficient (Lloyd et al., 2012). For instance, compared to adults, children experience a smaller number of motor units during volitional contraction which is reflected in relatively lower power production (Grosset et al., 2007). In addition, children demonstrate higher co-contraction of the Triceps surae and the Tibialis anterior muscles compared to adults (Lambertz et al., 2003). This excessive activation of antagonists increases the demands on the work of agonist muscles and decreases power output (Frost et al., 2002). Moreover, children rely more on reactive mechanisms, while adults utilize feedforward mechanisms to increase the level of muscle activity before contacting the limb with the surface (Oliver and Smith, 2010; Read et al., 2016). In addition, children’s muscle-tendon units undergo structural changes influencing SSC capability and increasing strength production during both phases of SSC (O’Brien et al., 2010).

Our findings are also in line with the previous work suggesting improvement of SSC performance with chronological age and maturation in youth until post puberty (Lloyd et al., 2012; Di Giminiani and Visca, 2017). In a recent study looking at SSC changes growth and maturation determined by RSI in youth female figure skaters (Lehnert et al., 2022), a gradual improvement in reactive strength from 8 to 13 years was observed, with significantly higher RSI values observed only in 11, 12 and 13-year-old participants compared to 8 and 9-year-olds and in 13-year-old participants compared to 10-year-olds. Non-linear improvements of the RSI were also reported among schoolboys aged 9, 12, and 15 years (Lloyd et al., 2012), with higher RSI seen in both 12 and 15 years of age compared to 9 years.

The apparent improvement in RSI with age is not consistent. Laffaye et al. investigated nonathletic boys and girls ages 11–20 and reported no significant change in RSI between age groups before the age of 13 in either sex (Laffaye et al., 2016). This conflicting finding may stem primarily from different fitness level of the measured groups, and from differences in the study methodology. However, Laffaye et al. also observed a progressive change with age in absolute leg stiffness, except for 17–18-year-old girls (Laffaye et al., 2016). This result is consistent with the notion that increases in body mass because of puberty will lead to increases in absolute leg stiffness in order to maintain true spring-mass model behaviour during ground contact (Granata et al., 2002). An increase in leg stiffness not only reduces the likelihood of excessive load of the knee passive structures, such as the ACL, particularly during dynamic movements (Hughes and Watkins, 2006) but also helps to produce efficient and powerful movement (Martin-Garetxana et al., 2024). Thus, the gradual improvement of RSI and leg stiffness with age observed in our study should be considered positive from the point of view of both injury risk of players and long-term performance development (Flanagan and Comyns, 2008; Müller et al., 2017).

Our results demonstrate a positive relationship between Flanker interference effect and dynamic balance, particularly in the non-dominant leg when either chronological or biological age were accounted for (Supplementary Table S2). A similar trend was observed in the dominant leg as well, albeit non-significant. The notion of limb dominance and balance is one of debate given that the dominant leg is typically defined as the preferred leg used to kick a ball (Markström et al., 2021a; Grinberg et al., 2022). This suggests that the non-dominant leg may be the one primarily used for dynamic stabilization, as it supports the body during open kinetic tasks of the dominant leg. It is therefore not surprising that during single-leg squats, we found that it was the non-dominant limb that demonstrated a significant relationship to Flanker interference effect. The link between postural control and cognitive abilities is supported by systematic neuroimaging evidence (Wittenberg et al., 2017), indicating that the cortex plays an active role in continuously sustaining and restoring postural balance in both static and dynamic tasks. Maintaining balance requires constant adjustments (Winter et al., 1996) and the suppression of unwanted movements. In this sense, individuals with better attentional control might be better equipped to make these adjustments and avoid dynamic instability. This relationship has implications for real-life situations, during which performing multiple simultaneous tasks while maintaining balance is common (Huxhold et al., 2006), making response inhibition ability crucial for controlling injury risk (Culvenor et al., 2016), particularly in complex situations and demanding environments such as team sport competitions. In this context, it has been proposed that athletes often utilize a “posture-first strategy” during postural-threatening situations, during which postural control is prioritized over an execution of cognitive tasks (Mohammadi-Rad et al., 2016). As such, real-life sports situations might place individuals with limited balance ability at risk for neurocognitive errors. While our chosen single-leg squat task was admittedly challenging in terms of both physical effort and balance, it did not involve a dual-task condition requiring attentional control, and thus the practical implications of the observed association remain speculative. Nevertheless, the observed association between Flanker interference effect and CoP antero-posterior ROM suggests that cognitive control mechanisms may still contribute to postural stability even in single-task conditions. Importantly, given the nature of the analysis implemented in the present study, the observed relationship reflects a mere association, and any causal relationship cannot be inferred. Moreover, Flanker interference effect accounted for approximately 16% of the variance in CoP anteroposterior range of motion. While this indicates a meaningful association, it also suggests that other factors contributed to the variability of this measure. Notably, no association was observed between attentional control and CoP medio-lateral ROM, most likely due to the task involving primarily sagittal movement. Conversely, frontal and transversal plane movements are more relevant in, e.g., lateral hops (Markström et al., 2021b), and in the context of knee joint stabilization and ACL injury risk (Hewett et al., 2005; Paterno et al., 2010). Same as the LESS, which incorporates dynamic knee valgus into its scoring criteria and similarly showed no association with attentional control, when age was not accounted for.

The overall median LESS score of 5.7 in our cohort aligns with previous findings, where scores above 5 are commonly reported in female athletes (Hanzlíková et al., 2021a). According to Padua et al., athletes with a LESS score of 5 or more had a 10.7 times higher risk of suffering an ACL injury compared to those scoring fewer than 5 errors (Padua et al., 2015). Therefore, the scores observed in our cohort suggest an elevated risk of ACL injury in young female volleyball players, which aligns with the literature reporting a high prevalence of ACL injuries in female volleyball athletes–significantly higher than that observed in their male counterparts (Loes et al., 2000).

In our study, the LESS score was not significantly associated with performance on the Eriksen Flanker test. The double-leg landing task assessed by LESS may be a fundamental motor skill, highly automated in volleyball players and requiring minimal conscious control or cognitive engagement. The absence of a reactive component in the task further reduces its cognitive demands. This aligns with findings from a study using the Movement-Specific Reinvestment Scale, which measures the propensity for conscious monitoring and control of movement–albeit a different aspect of cognitive control than that assessed by the Flanker Test–which also found no association with LESS scores (Hanzlíková et al., 2021b). The LESS as a clinical tool, has been shown to be suitable for assessing performance under cognitive load in adult females, as it can detect increased number of landing errors when a cognitive dual task is introduced (McWethy et al., 2025). However, it has a clear focus on gross movement patterns and aggregates all movement errors into a single total score. As such, the LESS may lack the sensitivity needed to detect links with attentional control and response inhibition, as assessed in our study. Future research should consider the use of three-dimensional motion capture systems, ideally combined with electromyography, to more precisely investigate whether distinct movement patterns, muscle activation level and timing are associated with the cognitive functions assessed by the Flanker test.

Several limitations should be acknowledged, with the most significant being the wide age range of the participants (7–15 years) which introduces a substantial developmental variability. This resulted in a heterogeneous cohort, where both chronological and biological age may have significantly influenced some of the biomechanical measures. While we accounted for both age and percentage of predicted adult height statistically using partial Spearman’s rank correlation, this approach—while appropriate for our study aims and sample size—does not allow for a detailed exploration of age-related interactions or nonlinear developmental effects. Given these factors, future research should consider narrower age groups to further reduce developmental variability and better isolate the relationship between attentional control and injury-related biomechanics. A second limitation is the absence of a dual-task condition, particularly during dynamic balance testing. While the observed association between Flanker interference effect and CoP antero-posterior ROM suggests a meaningful relationship, real-world situations often demand simultaneous cognitive and motor processes (Gondwe et al., 2020). The inclusion of dual-task paradigms in future research could provide deeper insights into how attentional control interacts with postural regulation in complex environments, such as sports competitions, where cognitive demands and balance adjustments occur concurrently (Hughes and Dai, 2021). Finally, given the cross-sectional nature of our study, the causal relationship between the outcomes cannot be inferred. Longitudinal studies are needed to further investigate potential causal links between attentional control and dynamic balance, as well as their practical implications.

## Conclusion

We explored the relationship between attentional control and injury-related biomechanics in young female volleyball players. While a significant relationship was observed for both reactive strength and lower-limb stiffness, it was likely influenced by the participants’ chronological age. When controlling for age, only the association between Flanker interference and an antero-posterior CoP movement of the non-dominant leg remained significant. This suggests that, among the injury-related biomechanical measures assessed, dynamic balance may have the strongest link to neurocognitive function in this population. Sport practitioners are advised to consider coupling dynamic stability exercises with neurocognitive evaluations for more holistic prevention of injuries in young athletes.

## Data Availability

The raw data supporting the conclusions of this article will be made available by the authors, without undue reservation.
