# Supplementary material for: The relationship between attentional control and injury-related biomechanics in young female volleyball players
Source: Front Physiol. 2025 Jul 4;16:1622026. doi: 10.3389/fphys.2025.1622026 (PMC12271222; doi:10.3389/fphys.2025.1622026)
Supplement: Supplementary file 1 [file Supplementaryfile1.docx]

Supplementary Material – Correlations with age

**Table S-1.** Spearman´s rank correlation between chronological age and biomechanical risk factors.

| **Correlated variables** | **Age** | |  |  |
| --- | --- | --- | --- | --- |
|  | r_s_ | P-value (FDR) |  |  |
|  |  |  |  |  |
| **Flanker interference effect (ms)** | -0.12 | 0.476 |  |  |
| **Congruent RT (ms)** | **-0.69** | **<0.001** |  |  |
| **Incongruent RT (ms)** | **-0.66** | **<0.001** |  |  |
| **LESS (errors)** | -0.11 | 0.455 |  |  |
| **Balance dominant leg** |  |  |  |  |
| **ROM CoP ML (cm)** | -0.23 | 0.122 |  |  |
| **ROM CoP AP (cm)** | -0.07 | 0.636 |  |  |
| **Speed CoP ML (cm/s)** | **-0.31** | **0.031** |  |  |
| **Speed CoP AP (cm/s)** | -0.12 | 0.476 |  |  |
| **Balance non-dominant leg** |  |  |  |  |
| **ROM CoP ML (cm)** | -0.09 | 0.540 |  |  |
| **ROM CoP AP (cm)** | 0.00 | 0.999 |  |  |
| **Speed CoP ML (cm/s)** | **-0.31** | **0.033** |  |  |
| **Speed CoP AP (cm/s)** | -0.05 | 0.719 |  |  |
| **Bilateral Leg Stiffness 2.2 Hz (kN/m)** | **0.74** | **<0.001** |  |  |
| **Dominant Leg Stiffness 2.5 Hz (kN/m)** | **0.76** | **<0.001** |  |  |
| **Nondominant Leg Stiffness 2.5 Hz (kN/m)** | **0.71** | **<0.001** |  |  |
| **Reactive strength index** | **0.66** | **<0.001** |  |  |

AP, antero-posterior direction; CoP, centre of pressure; LESS, Landing Error Scoring System; ML, medio-lateral direction; P-value (FDR), P-values corrected by False Discovery Rate method for 15 correlations presented; ROM, range of motion; RT, reaction time

**Table S-2.** Partial Spearman´s rank correlation between Flanker test performance and risk factors controlling for percentage of predicted adult height estimated by the Khamis-Roche method.

| **Correlated variables** | **Congruent RT** | |  | **Incongruent RT** | |  | **Flanker interference effect** | |
| --- | --- | --- | --- | --- | --- | --- | --- | --- |
|  | r_s partial_ | P-value (FDR) |  | r_s partial_ | P-value (FDR) |  | r_s partial_ | P-value (FDR) |
| **LESS (errors)** | 0.12 | 0.698 |  | 0.13 | 0.699 |  | 0.13 | 0.698 |
| **Balance dominant leg** |  |  |  |  |  |  |  |  |
| **ROM CoP ML (cm)** | -0.07 | 0.863 |  | -0.05 | 0.865 |  | 0.10 | 0.758 |
| **ROM CoP AP (cm)** | 0.21 | 0.460 |  | 0.30 | 0.241 |  | 0.32 | 0.187 |
| **Speed CoP ML (cm/s)** | 0.08 | 0.847 |  | 0.06 | 0.865 |  | 0.06 | 0.865 |
| **Speed CoP AP (cm/s)** | 0.04 | 0.865 |  | 0.06 | 0.865 |  | 0.11 | 0.758 |
| **Balance non-dominant leg** |  |  |  |  |  |  |  |  |
| **ROM CoP ML (cm)** | -0.14 | 0.688 |  | -0.02 | 0.899 |  | 0.25 | 0.366 |
| **ROM CoP AP (cm)** | -0.04 | 0.898 |  | 0.13 | 0.865 |  | **0.43** | **0.026** |
| **Speed CoP ML (cm/s)** | -0.02 | 0.899 |  | 0.02 | 0.899 |  | 0.16 | 0.594 |
| **Speed CoP AP (cm/s)** | 0.11 | 0.758 |  | 0.20 | 0.460 |  | 0.24 | 0.367 |
| **Bilateral Leg Stiffness 2.2 Hz (kN/m)** | -0.32 | 0.187 |  | -0.20 | 0.460 |  | 0.16 | 0.611 |
| **Dominant Leg Stiffness 2.5 Hz (kN/m)** | -0.24 | 0.367 |  | -0.18 | 0.499 |  | 0.07 | 0.864 |
| **Nondominant Leg Stiffness 2.5 Hz (kN/m)** | -0.24 | 0.367 |  | -0.20 | 0.460 |  | 0.02 | 0.899 |
| **Reactive strength index** | **-0.45** | **0.020** |  | **-0.48** | **0.020** |  | -0.22 | 0.433 |

AP, antero-posterior direction; CoP, center of pressure; LESS, Landing Error Scoring System; ML, medio-lateral direction; P-value (FDR), P-values corrected by False Discovery Rate method for 13 correlations presented; ROM, range of motion; RT, reaction time
